# Preparation and Characterization of Ultrasonically Modified Peanut Protein–Guar Gum Composite Emulsion Gels for 3D Printing

**DOI:** 10.3390/gels10120828

**Published:** 2024-12-14

**Authors:** Hong-Yan Yan, Shao-Bing Zhang

**Affiliations:** College of Food Science and Engineering, Henan University of Technology, Zhengzhou 450001, China; yanhongyanz@163.com

**Keywords:** peanut proteins, guar gum, emulsion gels, 3D printing, ultrasonic modification

## Abstract

This study aimed to prepare ultrasonically modified peanut protein–guar gum composite emulsion gels for 3D printing. The composition of the composite emulsion gels was determined in single-factor and orthogonal experiments. The results revealed that the optimal composite emulsion gels consisted of 6% peanut protein, 50% oil and 0.2% guar gum. After crushing pretreatment for 45 s, the printing deviation of the composite emulsion gels was reduced to 8.58 ± 0.20%. Moreover, after ultrasonic treatment (200 W for 20 min) of peanut proteins, the obtained composite emulsion gels presented the highest yield stress, hardness and G’ values, as well as a denser and more homogeneous microstructure. After protein ultrasonic modification (200 W or 600 W for 20 min), the printing accuracy and self-supporting properties of the composite emulsion gels for printing complex shapes significantly improved, which was attributed to their stronger textural and rheological properties; however, ultrasonically modified peanut protein–guar gum composite emulsion gels were not suitable for printing products with smooth surfaces.

## 1. Introduction

Three-dimensional food printing is an additive manufacturing technology that uses edible materials to build complex three-dimensional structures layer-by-layer based on digital models. It offers the advantages of flexible nutritional design, a simplified food supply chain and personalized model building [1]. However, the effective development of such materials still faces several challenges, as 3D food printing requires both excellent printability (extrusion flow) and formability (malleability) of the printing material (i.e., ink) [2]. In recent years, both high internal phase emulsions (HIPEs) (oil content > 74%) and emulsion gels have been widely used as 3D food printing materials because of their mechanical properties [3,4]. Compared with HIPEs, emulsion gels are semisolid gel materials with a three-dimensional network structure containing less oil, which is better for human health [5,6]. Moreover, HIPEs exhibit weak self-supporting behavior after 3D printing [7].

As natural amphiphilic macromolecules, peanut proteins have good emulsification properties, which makes them ideal substances for the preparation of protein emulsion gels. In our previous study, various peanut protein cold-set emulsion gels were induced with CaCl_2_, transglutaminase (TGase) or gluconic–delta–lactone (GDL) [8]. We further reported that GDL-induced emulsion gels prepared from peanut proteins pretreated with ultrasound (US) presented improved textural properties [9], which may have great potential for use in 3D printing. Many food protein emulsion gels, such as soybean protein isolate (SPI) [10], whey protein isolate (WPI) [11], walnut proteins [12], tea proteins [13] and zein [14], have been prepared and used for 3D printing. Nevertheless, to the best of our knowledge, the application of peanut protein emulsion gels for 3D food printing has not been reported.

Polysaccharides are often used to enhance the rheological properties and 3D printability of protein emulsion gels [12,15,16]. Guar gum (GG) is a nonionic galactomannan extracted from the endosperm of Cyamopsis tetragonolobus that functions in emulsification, thickening and stability [17]. It can be used to regulate the rheological and gelling properties of food materials to obtain suitable materials for 3D printing. Yu et al. [18] improved the network structure and 3D printing performance of SPI–polysaccharide composite emulsion gels by adding GG. At a GG content of 0.5% (*w*/*w*), the 3D-printed products demonstrated low-dimensional printing deviation with great self-supporting capability and smooth but slightly flawed surface texture. Li et al. [19] prepared emulsion gels for 3D printing using WPI, medium-chain triglycerides and GG and reported that 7% GG improved the 3D printing performance and significantly increased the printing accuracy and stability of WPI emulsion gels. Therefore, the addition of GG is expected to be beneficial for increasing the 3D printing accuracy of peanut protein emulsion gels.

In this work, peanut protein–guar gum (PP–GG) composite emulsion gels were prepared and used as 3D printing inks. First, the effects of protein content, oil fraction and GG content on the 3D printing properties of the composite emulsion gels were investigated. Next, based on the optimal emulsion gel composition, peanut proteins were treated with US, and composite emulsion gels with GG were prepared. The effects of US intensity on the textural properties, rheological properties, microstructure and 3D printing properties of composite emulsion gels were further investigated. This work provides a theoretical and methodological basis for the preparation of PP–GG composite emulsion gels suitable for 3D printing, thereby increasing the value of peanut proteins and GG for integrated use.

## 2. Results and Discussion

### 2.1. Effects of the Chemical Composition of the PP–GG Composite Emulsion Gels on Their 3D Printing Properties

#### 2.1.1. Effects of Protein Content on 3D Printing Properties

**Figure 1:**
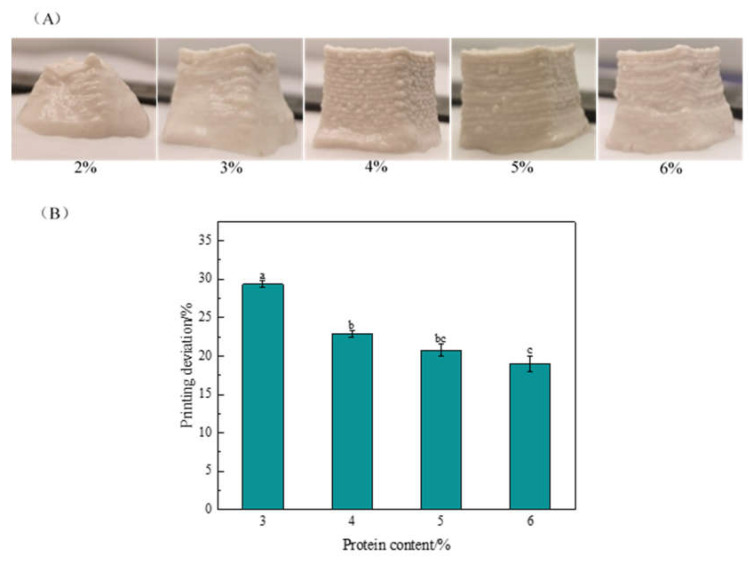
Effect of peanut protein content on 3D printing appearance (**A**) and printing deviation (**B**). The oil fraction was 30% and the GG content was 0.1%. The results in (**B**) are expressed as the means ± SD. Bars with different letters indicate significant differences (*p* < 0.05).

The effects of protein content on 3D printing properties are shown in Figure 1, with the oil phase content at 30% and the GG content at 0.1%. When the protein content was 2%, the material was too fluid and not plastic enough, which led to the severe collapse of the bottom of the printed product and failure to maintain the complete shape (Figure 1A). With increasing protein content, the material became progressively stiffer, less fluid, and more malleable, and the printed product had a more complete shape and clear contours with decreasing printing deviation (Figure 1B). However, when the protein content further increased to 6%, there were no significant (*p* < 0.05) changes in the printing properties. This may be attributed to the fact that an appropriate increase in protein content resulted in significant increases in the interactions between protein molecules [20], which resulted in an emulsion gel with higher stiffness that therefore exhibited greater self-supporting properties in 3D printing. Zhang et al. [21] reported that as the concentration of sea bass protein (SBP) microgel particles increased, the stacked layers of the HIPP emulsion were more easily distinguishable during the printing process, and the printed graphics with an SBP microgel particle concentration of 4% (*w*/*w*) had a clear contour and good print shape.

#### 2.1.2. Effects of the Oil Fraction on 3D Printing Properties

**Figure 2:**
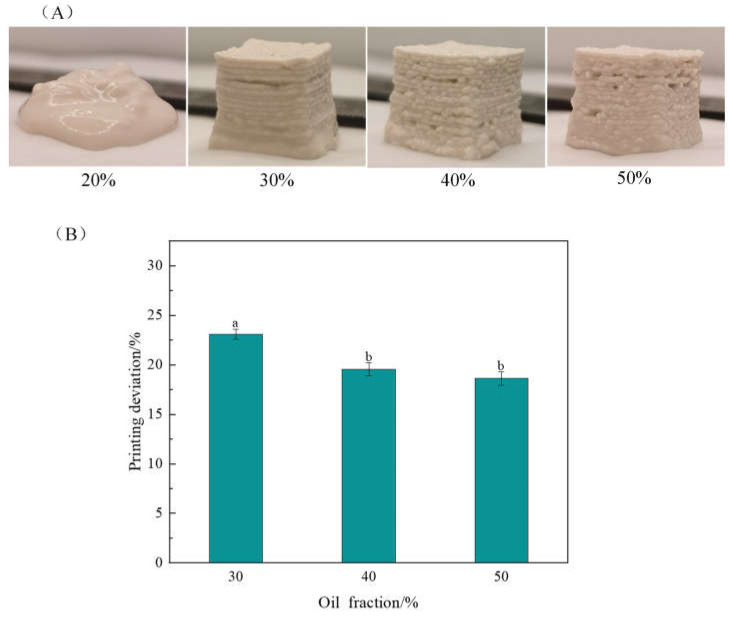
Effect of oil fraction on 3D printing appearance (**A**) and printing deviation (**B**). The protein content was 4% and the guar gum content was 0.1%. The results in (**B**) are expressed as the means ± SD. Bars with different letters indicate significant differences (*p* < 0.05).

The effects of oil phase content on 3D printing properties are shown in Figure 2, with the protein content at 4% and the GG content at 0.1%. When the oil fraction was 20%, the material flow was good, but the bottom of the printed product collapsed poorly and could not maintain its complete shape (Figure 2A). As the oil fraction increased, the printed product had a good printed shape, but the stacked layers appeared to be extruded discontinuously during the printing process, and the product had a rough surface. These effects are closely related to the reduction in the moisture content of the emulsion gels. The emulsion gels became hard and difficult to extrude. Relevant studies have shown that a slightly higher moisture content favors printing performance and the formation of a smooth structure [22].

As shown in Figure 2B, the printing deviation decreased significantly (*p* < 0.05) when the oil fraction was increased; this may be because oil acts as an “active filler” in the gel network structure and enhances the interaction with the gel matrix, resulting in a denser network structure and better viscoelastic emulsion gels [23]. Liu et al. [24] prepared gel-like emulsions from WPIs and soybean oil by microfluidization. When the oil fraction was further increased to 60%, the gel-like emulsions exhibited stronger semisolid behavior and were more suitable as 3D printing materials. In the present work, there was no significant (*p* < 0.05) change in the printing deviation when the oil fraction increased to 50%.

#### 2.1.3. Effects of the GG Content on 3D Printing Properties

**Figure 3:**
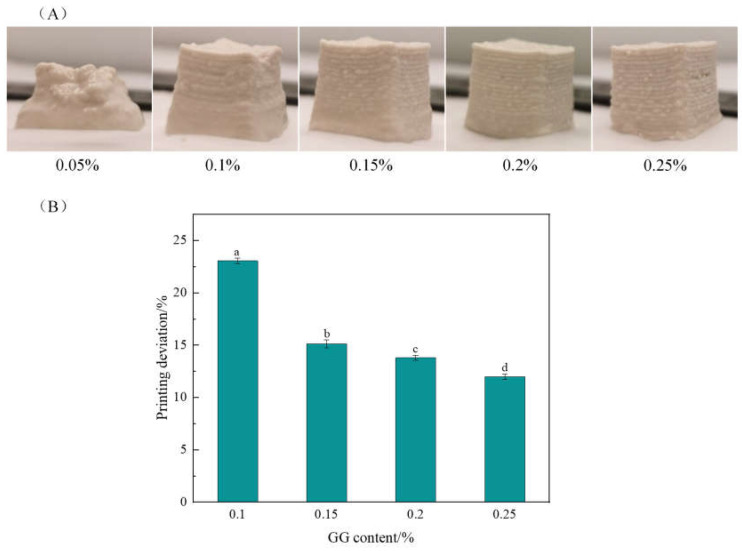
Effect of GG content on 3D printing appearance (**A**) and printing deviation (**B**). The protein content was 4% and the oil volume fraction was 30%. The results in (**B**) are expressed as the means ± SD. Bars with different letters indicate significant differences (*p* < 0.05).

The effects of the GG content on the 3D printing properties are shown in Figure 3, with the protein content at 4% and the oil phase content at 0.1%. When the GG content was 0.05%, the material was too fluid and not plastic enough, and the printed product did not have a complete shape. With increasing GG content, the 3D printing effect of the products improved, and all of them had good printed shapes (Figure 3A); at the same time, the printing deviation significantly (*p* < 0.05) decreased, and it was the lowest at a GG content of 0.25% (Figure 3B); this may be because the increase in polysaccharide concentration promoted the interaction of adjacent protein molecules. Additionally, a large number of hydroxyl groups in the main chain of GG combined with water and protein molecules by hydrogen bonds, thus increasing the strength of the gel matrix [19,25]. Furthermore, the neutral polysaccharide GG thickened the system to prevent oil droplets from coalescing, thus enhancing the network structure of the emulsion gels. Yu et al. [18] reported that the 3D printing accuracy of emulsion gels increased with increasing concentrations of xanthan or GG, which was mainly attributed to the emulsion gel having stronger gel network structures and higher viscosity.

#### 2.1.4. Orthogonal Experiments

The protein content, oil fraction, and GG content were used as factors in orthogonal experiments with the L9 (3^3^) orthogonal table, and printing deviation was used as an indicator. The results of the orthogonal experiments are shown in Table 1, and among the nine groups of experimental protocols, the printing deviation of the products was as low as 11.52% and as high as 18.38%. The analysis of extreme deviation revealed that the degree of influence of each factor on the printing deviation of emulsion gel was as follows: oil fraction > protein content > GG content. The best experimental combination for 3D printing was A_3_B_3_C_2_ (protein content: 6%; oil fraction: 50%; GG content: 0.2%), i.e., the ninth group experiment, which had the lowest printing deviation.

However, the surface of the printed products remained obviously rough under optimal experimental conditions. To improve this phenomenon, the emulsion gels were crushed before printing [26]; crushing (45 s) improved the appearance of the printed product and significantly (*p* < 0.05) reduced the printing deviation of the PP–GG composite emulsion gels (from 11.52 ± 0.34% to 8.58 ± 0.20%); this may be because crushing reduced the number of particles in the emulsion gels, making the emulsion gels more homogeneous and smoother, thereby reducing the degree of printing deviation.

### 2.2. Effects of US Modification of Peanut Proteins on the Physical Properties of PP–GG Composite Emulsion Gels

To further improve the textural properties of the PP–GG composite emulsion gels, peanut proteins were subjected to US treatment at different intensities, and the modified PP–GG composite emulsion gels were then prepared according to the above optimal experimental results (Table 1).

#### 2.2.1. Textural Properties

**Figure 4:**
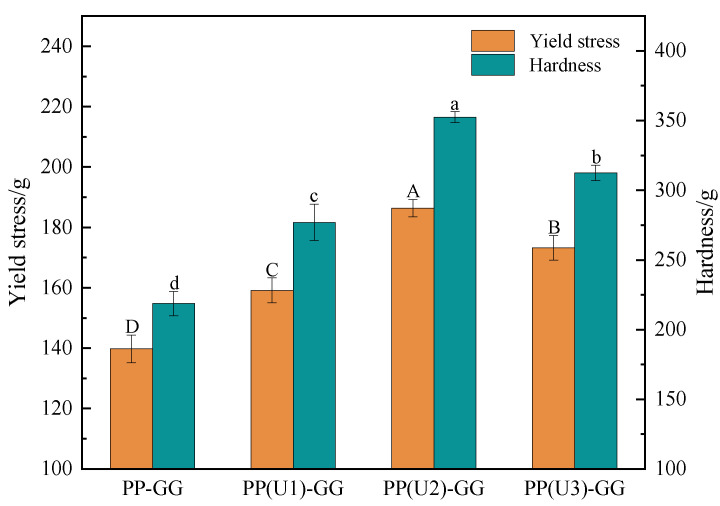
Effect of US modification on the textural properties of PP–GG composite emulsion gels. PP–GG: peanut protein–guar gum; PP(U1)–GG: PP–GG with low-intensity ultrasound modification (200 W/5 min); PP(U2)–GG: PP–GG with medium-intensity ultrasound modification (200 W/20 min); PP(U3)–GG: PP–GG with high-intensity ultrasound modification (600 W/20 min). The results are expressed as the means ± SD. Bars with different letters indicate significant differences (*p* < 0.05).

The yield stress is the force that causes the internal structure of the gel to begin to rupture [27], which reflects the hardness of the gel and is an important indicator for characterizing the extrudability and self-supporting properties of emulsion gels. As shown in Figure 4, the yield stress and hardness of the composite emulsion gels prepared from peanut proteins modified by US improved to different degrees. The yield stress and hardness of the emulsion gel reached the highest values (186.40 ± 2.87 g and 352.51 ± 3.85 g, respectively) at the medium-intensity (200 W, 20 min) US modification; this may be attributed to the increase in exposed hydrophobic and free sulfhydryl groups of peanut proteins after US modification [28]. The stronger hydrophobic interactions and greater number of disulfide bonds among protein molecules improved the emulsion gel textural properties. However, high-intensity (600 W, 20 min) US modification caused decreases in the yield stress and hardness of the emulsion gel; this may be because the strong US cavitation effect disrupted the inter- or intramolecular interactions of the protein molecules, resulting in protein disaggregation, thereby leading to the weakening of the gel network structure. Cheng et al. [29] found that both mono-frequency and simultaneous dual frequency US pretreatment could improve the hardness of whey protein emulsion gel.

#### 2.2.2. Rheological Properties

**Figure 5:**
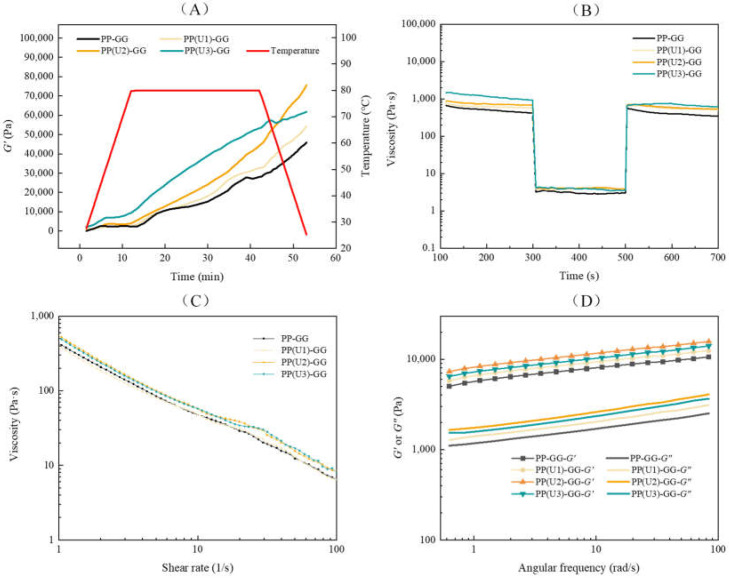
Effect of US modification on temperature scan (**A**), frequency scan (**B**), shear rate scan (**C**) and three interval thixotropy tests (**D**) of PP–GG composite emulsion gels. PP–GG: peanut protein–guar gum; PP(U1)–GG: PP–GG with low-intensity ultrasound modification (200 W/5 min); PP(U2)–GG: PP–GG with medium-intensity ultrasound modification (200 W/20 min); PP(U3)–GG: PP–GG with high-intensity ultrasound modification (600 W/20 min).

As shown in Figure 5A, the *G*′ values of all emulsion gels showed an increasing trend with time, indicating the gradual formation of the gel structure [30]. In the heating stage, the *G*′ value of the emulsion gel after high-intensity US modification increased more rapidly than those of the other samples. However, the *G*′ value of the emulsion gel after medium-intensity US modification became higher in the subsequent cooling stage, which was consistent with the results of the textural experiments (Figure 4). As shown in Figure 5B, all the samples after cooling presented higher *G*′ values than *G*″ values, indicating a highly interconnected network structure [31]. The *G*′ and *G*″ values of the emulsion gels increased after the peanut proteins were modified with US, indicating that US could unfold protein molecules and thereby facilitate gel formation [32]. The *G*′ and *G*″ values of the emulsion gel were the highest after medium-intensity US modification at various test frequencies. It has been demonstrated that emulsion gels with high *G*′ values ensure strong self-support for printed products and improve the printing accuracy of complex shapes [33,34]. In addition, the *G*′ values of all samples gradually increased with increasing frequency, indicating that these emulsion gels are mainly formed through noncovalent “physical crosslinks” [35], which are breakable or deformable.

As shown in Figure 5C, the viscosity of all the samples decreased significantly (*p* < 0.05) with increasing shear rate, indicating that the PP–GG emulsion gels were pseudoplastic fluids with shear thinning behavior. Compared with that of the untreated sample, the viscosity of the emulsion gel with low-intensity US modification did not significantly (*p* < 0.05) change at the same shear rate, whereas the viscosities of the emulsion gels with medium- and high-intensity US modifications significantly (*p* < 0.05) increased. Notably, if a material is chosen for 3D printing, its viscosity should be high enough to support layer-by-layer deposition of structures and to flow easily through nozzles with small diameters during extrusion [30,36], which facilitates the printing of complex shapes.

Three-interval thixotropic tests were performed on emulsion gels to assess their thixotropic recovery properties [37]. As shown in Figure 5D, the viscosity of all samples dropped sharply when the shear rate was increased from 1 s^−1^ to 100 s^−1^, indicating that the arrangement structure of the emulsion droplets in the gels was broken [38]. While restoring the shear rate to 1 s^−1^, the viscosity of all samples was greatly restored. Compared with the untreated sample, the emulsion gels after US modification exhibited similar thixotropic recovery properties. The results implied that the PP–GG emulsion gels could recover enough mechanical strength after passing through the nozzle to endure the loading of subsequent multiple layers.

#### 2.2.3. Microstructure

**Figure 6:**
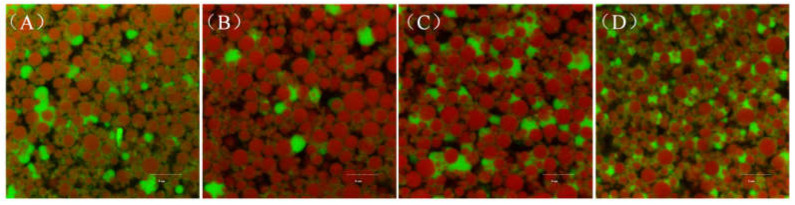
Effect of US modification on the microstructure of PP–GG composite emulsion gels. Typical CLSM images magnified at 400×, scale bar = 5 μm. (**A**): untreated PP; (**B**): 200 W, 5 min; (**C**): 200 W, 20 min; (**D**): 600 W, 20 min.

The microstructure of the composite emulsion gels was observed using confocal laser scanning microscopy (CLSM). As shown in Figure 6, red indicates oil droplets, and green indicates proteins. The oil droplets in the emulsion gels prepared from untreated peanut proteins were not homogeneous (Figure 6A). However, after medium- and high-intensity US modification, the oil droplets in the emulsion gels decreased in size and became homogeneous (Figure 6C,D). These findings suggest that US-modified proteins have better emulsifying activity in the preparation of O/W emulsions [35,39]. Compared with the control and low-intensity US-modified samples, the PP–GG emulsion gels had a dense and homogeneous protein gel network structure after medium- and high-intensity US modification (Figure 6C,D); this can be attributed to the formation of stronger chemical bonds within the gel, as mentioned in the discussion of textural properties. Usually, a dense gel network structure increases the viscosity of emulsion gels, improving the self-support of the printed products [36,37,40,41].

### 2.3. Effects of US Modification of Peanut Proteins on the 3D Printing Properties of PP–GG Composite Emulsion Gels

**Figure 7:**
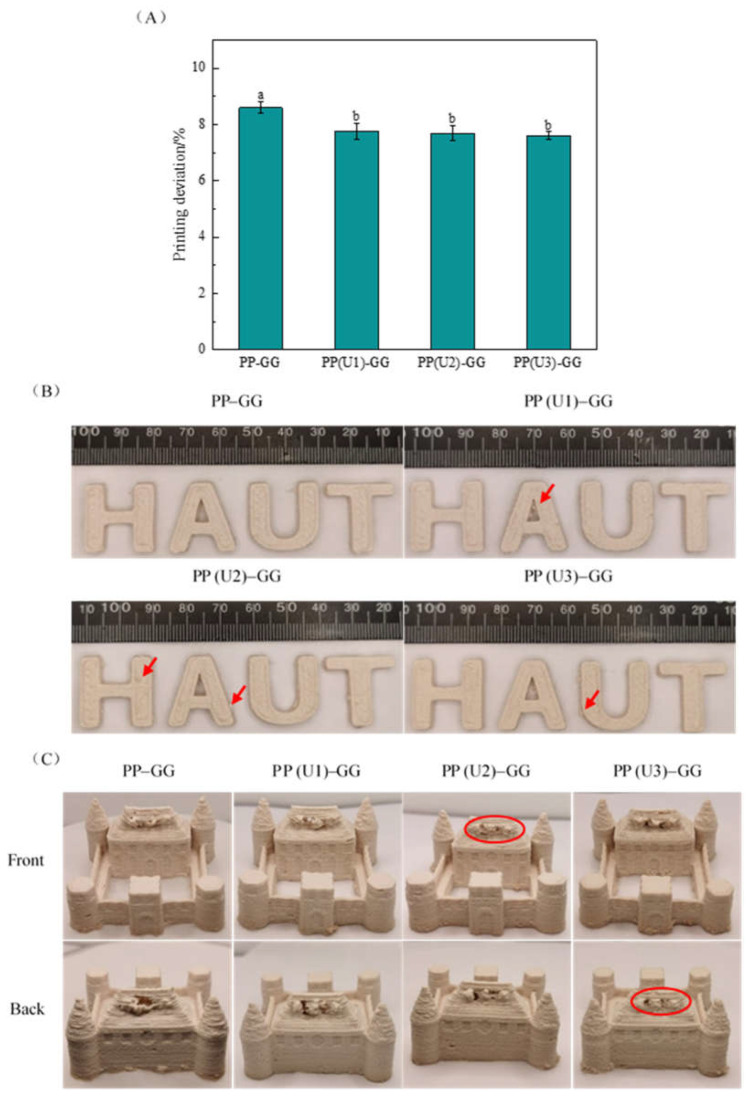
Effect of US modification on 3D printing deviation (**A**) and printing shapes ((**B**) “letter”; (**C**) “castle”) of PP–GG composite emulsion gels. PP–GG: peanut protein–guar gum; PP(U1)–GG: PP–GG with low-intensity ultrasound modification (200 W/5 min); PP(U2)–GG: PP–GG with medium-intensity ultrasound modification (200 W/20 min); PP(U3)–GG: PP–GG with high-intensity ultrasound modification (600 W/20 min). The results (**A**) are expressed as the means ± SD. Bars with different letters indicate significant differences (*p* < 0.05).

PP–GG composite emulsion gels prepared from untreated and modified peanut proteins were subjected to 3D printing. Figure 7A shows that the US treatment resulted in a significant (*p* < 0.05) reduction in the printing deviation of the emulsion gel, but there was no significant (*p* < 0.05) difference in the 3D printing deviation of the gels prepared from US-modified peanut proteins. Because the 3D printing properties of emulsion gels cannot be adequately reflected by measuring only the printing deviation, the various emulsion gels were further evaluated by printing a variety of structures. As shown in Figure 7B, the letters “HAUT” (Henan University of Technology) printed with all four emulsion gels exhibited uniform layer thicknesses and complete shapes and remained stable without collapsing. However, the letters “HAUT” printed by the three modified gels resulted in a slightly rough surface texture (red arrows in the figures). In general, emulsion gels with low yield stress are more favorable for extrusion during 3D printing, and the printed products have smooth textures [38,42]. However, US modification increased the mechanical strength (Figure 4) and viscosity (Figure 5A) of the emulsion gels; this may result in rougher lines during the printing process, thus reducing the smoothness of the printed products. Wang et al. [36,40] reported that when Pickering emulsion gels prepared from chitosan and glycyrrhizic acid-zein composite nanoparticles had high mechanical strength and viscosity, the printed products had a rough texture.

As shown in Figure 7C, for a more complex shape, such as the “castle” shape, all four emulsion gels can maintain the printed shape except for the top. The tops of the “castle” shapes printed by the untreated emulsion gels showed serious missing and collapsing phenomena, indicating the poor printing accuracy and self-supporting performance of the untreated gels. However, the tops of the “castle” shapes printed with the modified gels improved to different degrees. Specifically, after medium-intensity US modification, the emulsion gels printed the most complete detail on the top of the front side of the “castle” (circled in red in the figure); after high-intensity US modification, the emulsion gels printed the most complete detail on the top of the back side of the “castle” (circled in red in the figure). The results show that medium- and high-intensity US modification of peanut proteins can improve the printing accuracy of emulsion gels when complex shapes are printed. As discussed above, the emulsion gels after medium- and high-intensity US modifications had high hardness and viscosity, which resulted in a nonsmoothed surface when letters were printed. However, these modifications improved the printing accuracy and self-supporting properties of the printed products, thus benefiting the printing of complex shapes. US modification may also be able to improve the 3D printing properties of emulsion gels prepared from other globulin proteins because they share a similar structure to peanut proteins.

## 3. Conclusions

In this work, peanut protein emulsion gels with GG were prepared as 3D food printing materials. The optimum composition was as follows: 6% protein content, 50% oil phase and 0.2% GG content. After crushing for 45 s, the optimal printing deviation of the composite emulsion gels was 8.58 ± 0.20%. Moreover, after peanut proteins were treated with US, the composite emulsion gels presented stronger textural and rheological properties, as well as a denser and more homogeneous microstructure. The emulsion gels after medium- and high-intensity US modification were suitable for printing products with complex shapes and structures because they possessed stronger textural properties and thus were able to print the details of the product and possessed good self-supporting ability; however, if the structure of the products was relatively simple and required high surface smoothness, there was no need to perform US modification on peanut proteins. In summary, the PP–GG composite emulsion gels after US modification can be used as a material for the development of tailor-made 3D printing products for people with chewing and swallowing disparities, which can ensure that the 3D printing products have a soft texture as well as an exquisite appearance, and can also provide sufficient proteins.

## 4. Materials and Methods

### 4.1. Materials

Peanuts were obtained from the local market. Soybean oil was purchased from COFCO Yellow Sea Grain and Oil Industry Co., Ltd. (Rizhao, China). Guar gum was purchased from Henan Wanbang Chemical Technology Co., Ltd. (Zhengzhou, China). Gluconic–delta–lactone (GDL), Nile red and fluorescein isothiocyanate (FITC) were purchased from Shanghai McLean Biochemical Technology Co., Ltd. (Shanghai, China).

### 4.2. Extraction of Peanut Proteins

Peanut proteins were extracted according to the method of Jiang, Zhang, Zhang, and Peng [43]. In total, 300 g of unpeeled peanut kernels was removed and crushed in a new type of sealed crusher for 2 min. Then, 1800 mL of distilled water was added, mixed and dispersed homogeneously, and the pH was adjusted to 10.0. The mixture was placed in a water-bath oscillator and oscillated at 50 °C for 30 min (150 r/min). After that, centrifugation was performed for 15 min (3300× *g*), and the oil phase of the uppermost layer, the cream layer and the residue of the lowermost layer were discarded. The pH of the supernatant was adjusted to 4.5, and the mixture was washed twice with water and centrifuged for 15 min (3300× *g*). The precipitate, which was enriched with peanut proteins, was freeze-dried for 24 h and set aside.

### 4.3. Ultrasonic (US) Treatment of Peanut Proteins

Peanut protein solution was stirred overnight at room temperature, the pH was adjusted to 7.0, and the peanut protein solution (25 mL) was placed in a 100 mL beaker for modification with a US processor (Scientz-IID, NingBo Scientz Biotechnology Co., Ltd., Ningbo, China) with a 0.636 cm diameter titanium probe (without temperature control). The ultrasound treatment conditions were 200 W for 5 min, 200 W for 20 min, and 600 W for 20 min, respectively. The resulting samples were sequentially numbered PP (U1), PP (U2), and PP (U3).

### 4.4. Optimization of Preparation Conditions for PP–GG Composite Emulsion Gels for 3D Printing

#### 4.4.1. Preparation of PP–GG Composite Emulsion Gels

An appropriate amount of peanut protein was added to distilled water and stirred overnight at room temperature (pH 7) to obtain a peanut protein solution. After that, soybean oil (20–50%, *v*/*v*) was added to the peanut protein mixture (2–6%, *w*/*v*). The mixture was processed using a digital high-speed homogenizing and dispersing machine (FJ300-PSH, Shanghai Specimen Model Factory Co., Ltd., Shanghai, China) at 15,000 r/min for 2 min 20 s and then sonicated (20 min, pulse duration of 3 s and off time of 2 s, <35 °C) at 300 W to obtain a fine protein emulsion [44]. Finally, GG stock solution (0.05–0.25%, *w*/*v*) and GDL (0.2% and 0.3%, *w*/*v*, respectively) were added to the prepared protein emulsion, which was subsequently heated at 80 °C for 30 min and dispersed by high-speed shearing at 10,000 rpm for 2 min 30 s. The mixture was then immediately cooled in ice water and stored in the refrigerator at 4 °C overnight to obtain the PP–GG composite emulsion gels. Based on the results of the single-factor experiments, a three-factor and three-level orthogonal experiment (Table 1) was designed to optimize the conditions for the preparation of PP–GG composite emulsion gels suitable for 3D printing.

#### 4.4.2. Measurement of the Dimensional Printing Deviation of PP–GG Emulsion Gels

A cube with a side length of 15 mm was printed using PP–GG composite emulsion gels as the printing ink and was immediately stored in a freezer at −20 °C. After 24 h, a photograph of the bottom surface of the cube was taken, and a ruler with a length scale reference was used as needed. The dimensional printing deviation was subsequently quantified using the image analysis program ImageJ. The percentage deviation of the bottom surface of the cube from the expected perimeter was calculated as the print size deviation [44], referred to as printing deviation.

### 4.5. Preparation of Ultrasonically Modified PP–GG Composite Emulsion Gels

The ultrasonically modified PP–GG composite emulsion gels consisted of 6% (*w*/*v*) ultrasonically modified peanut proteins, 50% (*v*/*v*) soybean oil and 0.2% (*w*/*v*) GG, prepared as described in Section 4.4.1.

### 4.6. Characterization of Ultrasonically Modified PP–GG Composite Emulsion Gels

#### 4.6.1. Textural Properties

The emulsion gels were determined by puncture (probe P/0.5) using a physical property tester (TA-XTplusC, Stable Micro System Co., Ltd., Godalming, UK). To determine the yield stress and hardness of the emulsion gels, the pretest, posttest and speed parameters of the physical property tester were set to 1.0, 10.0 and 1.0 mm/s, the penetration distance was 20 mm, and the trigger force was 5 g.

#### 4.6.2. Rheological Properties

##### Dynamic Viscoelastic Measurement

The emulsion samples (1.5 mL) mixed with GDL and GG were placed on a 35 mm parallel plate of a Hacker rheometer (RS60, Haake Co., Ltd., Karlsruhe, Germany). The parallel plate gap was set to 1.0 mm. At a 1.0% strain and a fixed frequency of 1.0 Hz, the sample was heated from 25 °C to 80 °C at 5 °C /min and kept at 80 °C for 30 min, and then decreased from 80 °C to 25 °C at the same rate. The storage modulus (*G*′) was recorded during the heating and cooling cycle.

##### Rheological Properties of Emulsion Gels

All the prepared emulsion gels were relaxed at 25 °C for 30 min prior to rheological testing. Prior to testing, a two-minute equilibration time was used to eliminate load effects. A strain scan test was performed to determine the linear viscoelastic region (LVR).

(1) Flow behavior test

Stable shear tests were performed in the range of 1–100 s^−1^ to determine the viscosities (apparent viscosity) of these emulsion gels.

(2) Small-amplitude oscillatory shear (SAOS) test

Sweep tests were performed over a frequency range of 0.1 to 10 Hz to identify the storage/elastic modulus (*G*′) and loss/viscous modulus (*G*″) values of the emulsion gels. All tests were performed at a constant strain of 0.1%, which is within the LVR range [45].

(3) Three interval thixotropy tests (3ITT)

This test was used to simulate the 3D printing process and assessed the shear recoverability of the emulsion gels. The test was divided into three stages and a duration of 200 s in each stage. The first stage was to fix the shear rate at 1 s^−1^ to simulate the emulsion gels before printing; the second stage was to fix the shear rate at 100 s^−1^ to simulate the printing process of emulsion gels; the third stage was the same as the first step to simulate the state of the emulsion gels after 3D printing [38].

#### 4.6.3. Microstructure

The method used by Hou, Guo, Wang, and Yang [46] was adopted with slight modifications. Nile red (0.1%, *w*/*v*) and fluorescein isothiocyanate (FITC) (0.1%, *w*/*v*) were selected to stain the emulsions, which were used as fluorescent dyes for the oil phase (red) and protein phase (green), respectively. The emulsion gel was subsequently prepared according to the method described in Section 4.4.1. The excitation wavelengths of Nile red and FITC were set to 633 nm and 488 nm, respectively, and the microscopy images of the emulsion gels were obtained by confocal laser scanning microscopy (FV3000, Olympus Co., Ltd., Tokyo, Japan).

### 4.7. Printability of Ultrasonically Modified PP–GG Composite Emulsion Gels

The emulsion gels were printed using a 3D food printer (FOODBOT-S2Pro, Shiyin Technologies Co., Ltd., Hangzhou, China). Three-dimensional models were converted using the slicing software Cura and read by a 3D food printer. The 3D printing parameters were as follows: nozzle diameter, 0.84 mm; printing speed, 20 mm/s; layer height, 0.6 mm; and fill density, 90% [47]. The “AUTA” and “Castle” models were used to evaluate the printing accuracy and support capacity of the materials.

### 4.8. Statistical Analysis

All experiments were repeated at least twice; the data were processed using SPSS Statistics software (SPSS 22.0, IBM, Chicago, IL, USA) and are expressed as the means ± standard errors. In addition, the data were subjected to Duncan variance analysis with a significance level of *p* < 0.05, and Origin software (Origin 9.0, Originlab, Northampton, MA, USA) was used for graphing.

## Figures and Tables

**Table 1:** Orthogonal array design matrix L9 (3^3^) and experimental results for 3D printing deviation of PP–GG composite emulsion gels.

Experimental Group	Factors			Printing Deviation/%
	A: Protein Content/%	B: Oil Fraction/%	C: GG Content/%	
1	1 (4%)	1 (30%)	1 (0.15%)	18.38 ± 0.76
2	1	2 (40%)	2 (0.2%)	15.83 ± 0.99
3	1	3 (50%)	3 (0.25%)	13.96 ± 0.40
4	2 (5%)	1	2	17.34 ± 0.52
5	2	2	3	16.36 ± 0.36
6	2	3	1	12.55 ± 0.74
7	3 (6%)	1	3	15.89 ± 0.40
8	3	2	1	15.03 ± 0.44
9	3	3	2	11.52 ± 0.34
*K_1_*	48.17	51.61	45.96	
*K_2_*	46.25	47.22	44.69	
*K_3_*	42.44	38.03	46.21	
*R*	5.73	13.58	0.25	
*R* order	B > A > C			
Optimal conditions	A_3_B_3_C_2_			

All data are expressed as mean ± SD (*n* = 3). *K_i_*_(1,2,3)_ are the average printing deviation of each factor in each of the levels. *R* referred to the result of extreme analysis.

## Data Availability

The original contributions presented in this study are included in the article material. Further inquiries can be directed to the corresponding author.
